# TcMYB8, a R3-MYB Transcription Factor, Positively Regulates Pyrethrin Biosynthesis in *Tanacetum cinerariifolium*

**DOI:** 10.3390/ijms232012186

**Published:** 2022-10-12

**Authors:** Li Zhou, Jiawen Li, Tuo Zeng, Zhizhuo Xu, Jing Luo, Riru Zheng, Yuanyuan Wang, Caiyun Wang

**Affiliations:** 1 Key Laboratory for Biology of Horticultural Plants, Ministry of Education, College of Horticulture & Forestry Sciences, Huazhong Agricultural University, Wuhan 430070, China; 2 School of Life Sciences, Guizhou Normal University, Guiyang 550025, China

**Keywords:** function analysis, MYB transcription factors, pyrethrins, *Tanacetum cinerariifolium*

## Abstract

Pyrethrins are a mixture of terpenes, with insecticidal properties, that accumulate in the aboveground parts of the pyrethrum (*Tanacetum cinerariifolium*). Numerous studies have been published on the positive role of MYB transcription factors (TFs) in terpenoid biosynthesis; however, the role of MYB TFs in pyrethrin biosynthesis remains unknown. Here, we report the isolation and characterization of a *T. cinerariifolium* *MYB* gene encoding a R3-MYB protein, TcMYB8, containing a large number of hormone-responsive elements in its promoter. The expression of the *TcMYB8* gene showed a downward trend during the development stage of flowers and leaves, and was induced by methyl jasmonate (MeJA), salicylic acid (SA), and abscisic acid (ABA). Transient overexpression of *TcMYB8* enhanced the expression of key enzyme-encoding genes, *TcCHS* and *TcGLIP*, and increased the content of pyrethrins. By contrast, transient silencing of *TcMYB8* decreased pyrethrin contents and downregulated *TcCHS* and *TcGLIP* expression. Further analysis indicated that TcMYB8 directly binds to cis-elements in pro*TcCHS* and pro*TcGLIP* to activate their expression, thus regulating pyrethrin biosynthesis. Together, these results highlight the potential application of TcMYB8 for improving the *T. cinerariifolium* germplasm, and provide insight into the pyrethrin biosynthesis regulation network.

## 1. Introduction

Pyrethrum (*Tanacetum cinerariifolium*) is the largest insecticidal plant cultivated in the world, and has been commercialized and developed as a crop for the extraction of a group of natural insecticides, called pyrethrins, for more than 100 years [1,2]. Although synthetic pyrethroids have been successfully used to control insect populations, consumers prefer using natural pyrethrins as an environmentally friendly and harmless alternative [3]. The pyrethrin content of *T. cinerariifolium* is as high as 2.5% in dry flowers [4], much higher than that of *Tanacetum coccineum*, which has been historically used as a source of pyrethrins [5]. However, how to further improve the pyrethrin content of pyrethrum remains a hot topic of research because of the annual increase in the demand for pyrethrins in the international market [6]. Some studies indicate that high-yielding pyrethrum plants have been obtained through artificial selection, cross-breeding, polyploidy induction, and population improvement; however, these approaches are time-consuming and expensive and ineffective in maintaining the beneficial traits of plants [7,8,9]. Therefore, to further improve the production of pyrethrins, it is critical to explore the pyrethrin anabolic pathway and its regulatory mechanism.

Natural pyrethrins are a mixture of six terpene esters, and their biosynthetic pathway includes the formation of the acid moiety, followed by the synthesis of the alcohol moiety, and finally the esterification reaction [6]. During the biosynthesis of the acid moiety, two molecules of dimethylallyl pyrophosphate (DMAPP) are converted by chrysanthemol synthase (CHS) into chrysanthemic alcohol [10,11], which is then converted into chrysanthemic acid and pyrechric acid by alcohol dehydrogenase2 (ADH2) and aldehyde dehydrogenase1 (ALDH1) [12]. To form the alcohol group, linolenic acid is catalyzed by lipoxygenase (LOX), allene oxide cyclase (AOC), oxo-phytodienoic acid reductase (OPR), and other unidentified enzymes to form jasmone [13,14,15,16], which is then converted into the alcohol moiety by a P450 cytochrome oxidoreductase, namely jasmolone hydroxylase (JMH) and pyrethrone synthase (PYS) [17]. Finally, the acid and alcohol moieties are conjugated by a GDSL lipase-like protein (GLIP) to form pyrethrins [18]. CHS functions at the first branch point in the acid biosynthesis pathway, and overexpression of *TcCHS* in pyrethrum significantly increased the pyrethrin content [19]. GLIP functions at the last step of pyrethrin biosynthesis [18]. Our previous research reported that the binding affinity between the TcGLIP enzyme and substrate directly affects the pyrethrin content [20]. These findings suggest that CHS and GLIP are key enzymes in the pyrethrum biosynthesis pathway. Thus, regulating the expression of *CHS* and *GLIP* genes is expected to significantly affect pyrethrin biosynthesis.

MYB transcription factors (TFs) play an important role in the biosynthesis of secondary metabolites including alkaloids [21], phenylpropanoids [22,23], and terpenes [24]. MYB TFs regulate the expression of target genes by directly binding to the promoters [25,26,27] bounding MYB binding sites (MBS), L1-box and recognizing binding sites rich in A, C base [28,29,30]. Previous studies have implied that MYB TFs were involved in regulating the synthesis of terpene [27,31]. Pyrethrins, as a unique terpene anti-insect substance in pyrethrum, MYB TFs have not been reported to participate in the upstream synthesis regulation of pyrethrins. In this study, we cloned the *TcMYB8* gene and verified its role in regulating the expression of *TcCHS* and *TcGLIP* in pyrethrin biosynthesis. This study reveals the mechanism of pyrethrin biosynthesis and will serve as a guide for breeding pyrethrum cultivars with high pyrethrum content.

## 2. Results

### 2.1. Characterization of TcMYB8 and Its Promoter

**Figure 1:**
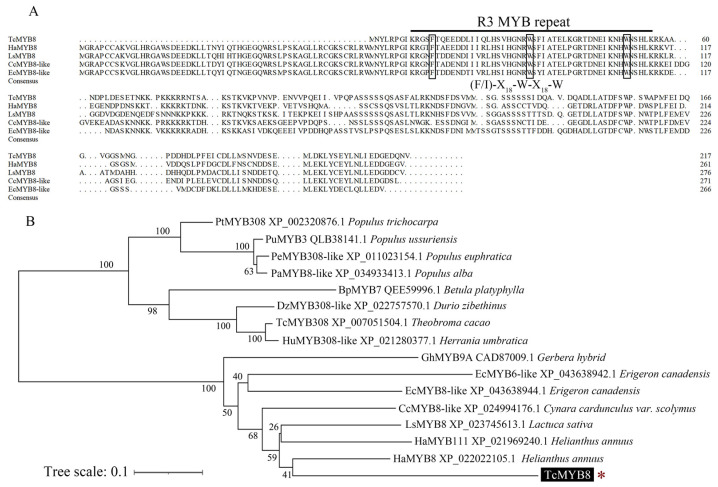
Multiple sequence alignment, conservative domain analysis, and phylogenetic analysis of TcMYB8 and other plant MYB proteins. (**A**) Multiple sequence alignment of the deduced TcMYB8 protein and MYB8 proteins of *Helianthus annuus*, *Lactuca sativa*, *Cynara cardunculus*, and *Erigeron canadensis*. Solid black line indicates the conserved DNA-binding domainR3 MYB repeat. (**B**) Phylogenetic analysis of TcMYB8 and MYB subfamily proteins from different plant species. Multiple sequence alignment was carried out using ClustalW, and the phylogenetic tree was constructed using MEGAX64 with the neighbor-joining method of 1000 bootstrap replications. * represents MYB8 protein in *T. cinerariifolium*.

Through transcriptome analysis, the *TcMYB8* gene was isolated from in vitro grown seedlings of *T. cinerariifolium* clone ‘W99′. The length of open reading frame (ORF) (GenBank accession no. OP087309) was 654 bp, and is predicted to encode a 217-amino acid protein, with a molecular weight of 24.26 kDa and an isoelectric point of 4.83. TcMYB8 contains only one conserved MYB domain (Figure S1), an R3 repeat ((F/I)-X_18_-W-X_18_-W) at the N terminus (Figure 1A), thus belonging to the 1R-MYB subfamily. MYB8s are involved in the response to phytophagous insects and the regulation of secondary metabolites. Multiple amino-acid sequence alignment indicated that TcMYB8 is highly similar to the MYB8 or MYB8-like proteins of Compositae family plants (Figure 1A). To investigate the evolutionary relationship between TcMYB8 and 15 other MYBs, a phylogenetic tree was constructed using the neighbor-joining method (Figure 1B). A blast phylogenetic analysis revealed that TcMYB8 was polymerized with sunflower (*Helianthus annuus*) MYB8 (HaMYB8).

A 935-bp upstream sequence of the *TcMYB8* coding region was isolated by genome walking, and cis-regulatory elements on the *proTcMYB8*region were identified using the PlantCARE database. Motifs known to play a role in secondary metabolism and in the response to biotic and abiotic stresses, light, and plant hormones, such as methyl jasmonate (MeJA), salicylic acid (SA), and abscisic acid (ABA), were found in the *TcMYB8* promoter (Table S1).

### 2.2. Expression Analysis and Subcellular Localization of TcMYB8

**Figure 2:**
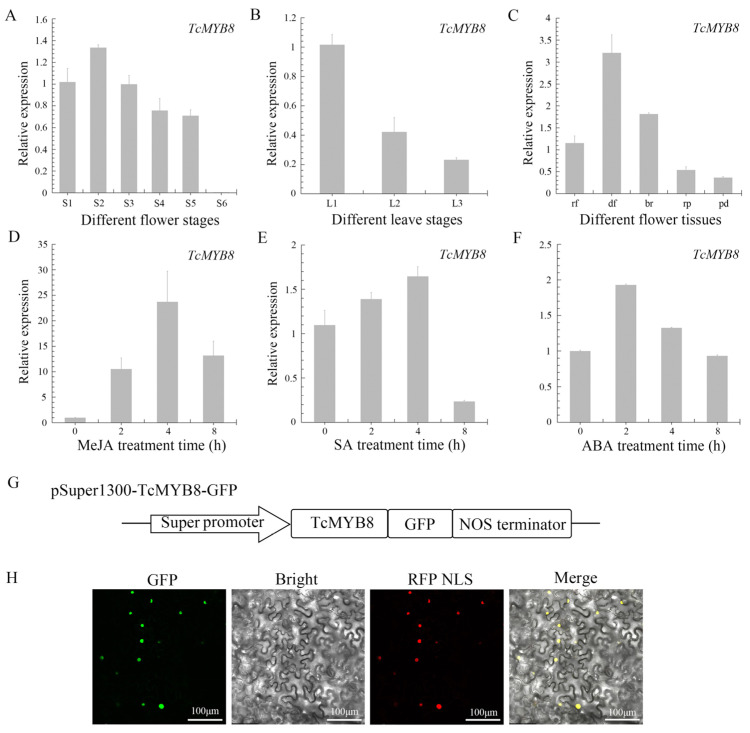
Expression analysis of the *TcMYB8* gene and subcellular localization of the encoded protein. (**A**,**B**) Analysis of *TcMYB8* expression by qRT-PCR in flowers (**A**) and leaves (**B**) at different developmental stages. S1–S6 and L1–L3 represent the different developmental stages of flowers and leaves, respectively. S1: bud stage; S2: half of the peripheral ray florets open; S3: 1st row of disk florets open; S4: half of the rows of disk florets open; S5: all rows of disk florets open; S6: past flowering; L1: small leaves (5–10 mm in width); L2: medium leaves (15–20 mm in width); L3: large leaves (>25 mm in width). (**C**) qRT-PCR analysis of *TcMYB8* expression in different tissues of S2-stage flowers; rf, ray florets; df, disk florets; br, bracts; rp, receptacle; pd, pedicel. (**D**–**F**) qRT PCR analysis of *TcMYB8* expression in tissue culture seedlings at 0, 2, 4, and 8 h after treatment with MeJA (**D**), SA (**E**), and ABA (**F**). (**G**) Diagram of the pSuper1300-TcMYB8-GFP construct. (**H**) Subcellular localization of TcMYB8 in *N. benthamiana* leaves. Bar = 100 μm.

To study the temporal and spatial expression patterns of *TcMYB8*, the expression levels of *TcMYB8* were determined in flowers and leaves at different developmental stages by quantitative real-time PCR (qRT-PCR). Among the different stages of flower development, the expression level of *TcMYB8* peaked at the S2 stage (Figure 2A). The *TcMYB8* gene was mainly expressed in small leaves (5–10 mm in width), and gradually decreased as the blade width reached the medium or large size (>15 mm) (Figure 2B). The *TcMYB8* gene was constitutively expressed in all flower parts, with a predominant expression in disk florets (Figure 2C). Moreover, the *TcCHS* and *TcGLIP* genes presented very similar expression trends as *TcMYB8* in temporal and spatial expression patterns (Figure S2A–C).

Consistent with the cis-elements in the *TcMYB8* promoter, the expression of *TcMYB8* was significantly altered after treatment with MeJA, SA, and ABA. *TcMYB8* transcripts accumulated rapidly after the MeJA treatment, peaking at 4 h, and then declined slowly (Figure 2D). With the SA and ABA treatment, the expression level of *TcMYB8* increased slightly (Figure 2E,F). These results suggest that the *TcMYB8* gene responds to MeJA, SA, and ABA.

To explore the localization of TcMYB8 in cells, the TcMYB8-GFP fusion was transiently expressed in tobacco (*Nicotiana benthamiana*) leaves. The TcMYB8-GFP signal completely coincided with the RFP-NLS nuclear marker, confirming its localization to the nucleus (Figure 2G). These results suggest that TcMYB8 potentially acts as a TF.

### 2.3. TcMYB8 Expression Promotes Pyrethrin Biosynthesis

**Figure 3:**
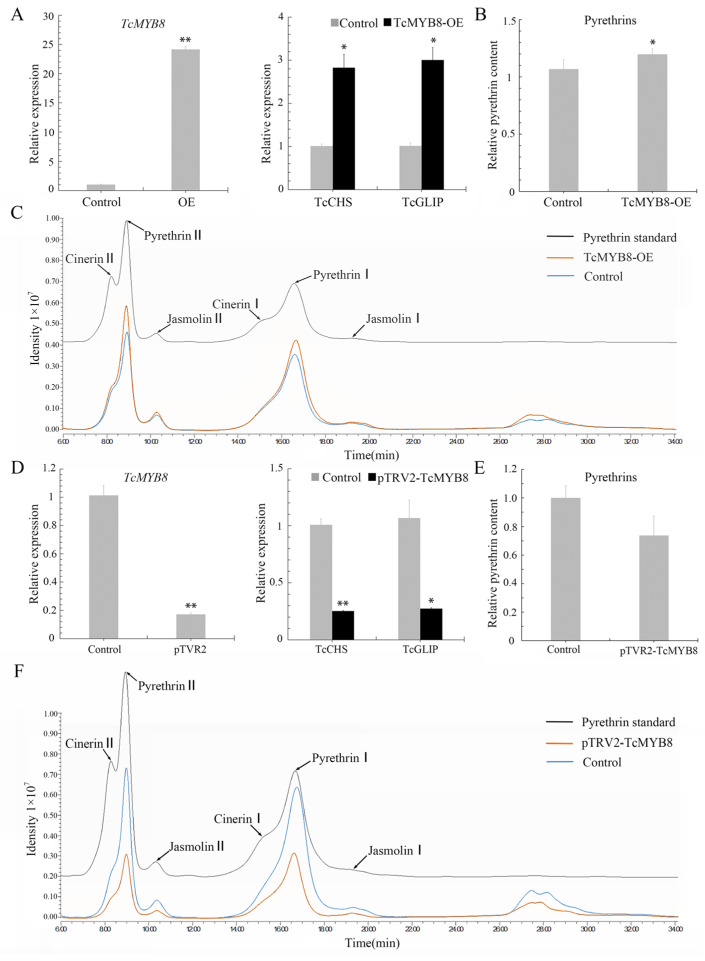
Functional characterization of TcMYB8 in *T. cinerariifolium* leaves. (**A**) Expression levels of pyrethrin biosynthetic genes in *T. cinerariifolium* leaves transiently expressing TcMYB8. Gene expression was detected by qRT-PCR at 4 days post-infiltration (dpi). (**B**) Relative pyrethrin content in *T. cinerariifolium* leaves after transient TcMYB8 overexpression at 4 days. (**C**) Contents of pyrethrin I and pyrethrin II in in *T. cinerariifolium* leaves transiently expressing TcMYB8. The ordinate represents the peak area detected by HPLC. The black arrows indicate the six major constituents of pyrethrins. Control: transient overexpression of the empty vector; TcMYB8-OE: transient overexpression of pGreen-62-SK-TcMYB8. (**D**) Expression levels of pyrethrin biosynthetic genes in *T. cinerariifolium* leaves expressing the Tobacco rattle virus (TRV) vector for silencing the TcMYB8 gene by virus-induced gene silencing (VIGS). Gene expression was detected by qRT-PCR at 14 dpi. (**E**) Relative pyrethrin content in *T. cinerariifolium* leaves after being silenced for TcMYB8 by TRV VIGS. (**F**) Contents of pyrethrin I and pyrethrin II in leaves silenced for TcMYB8 by TRV VIGS. The ordinate represents the peak area detected by HPLC. The black arrows indicate the six major constituents of pyrethrins. Control: transient expression of the pTRV2 empty vector and pTRV1; pTRV2-TcMYB8: transient expression of pTRV2-TcMYB8 and pTRV1. Values shown are the mean ± SE, for which asterisks indicate significance differences (two-tailed t-test, *: *p* < 0.05; **: *p* < 0.01).

The expression of TcMYB8 in *T. cinerariifolium* leaves was detected by qRT-PCR at 4 days post-infiltration. The transcript levels of TcMYB8, TcCHS, and TcGLIP were 24.1-, 2.8-, and 3.0-fold higher, respectively, in leaves infiltrated with TcMYB8 than in those infiltrated compared with control leaves (Figure 3A). Furthermore, we examined the contents of six pyrethrins in the transformed pyrethrum leaves by high-performance liquid chromatography (HPLC). The total pyrethrins increased to control leaves (Figure 3B), and the contents of pyrethrin I and pyrethrin II increased significantly (Figure 3C) Additionally, in the leaves of TcMYB8-RNAi lines, the expression level of TcMYB8 was lower than that in the control leaves, and the transcript levels of TcCHS and TcGLIP were respectively reduced to 0.25- and 0.27-fold in comparison with the control group (Figure 3D). The content of pyrethrins in leaves was lower than the control level (Figure 3E,F). Taken together, these results suggested that TcMYB8 upregulates the transcription of TcCHS and TcGLIP and promotes the synthesis of pyrethrins.

### 2.4. TcMYB8 Directly Binds to the Promoters of TcCHS and TcGLIP Genes

**Figure 4:**
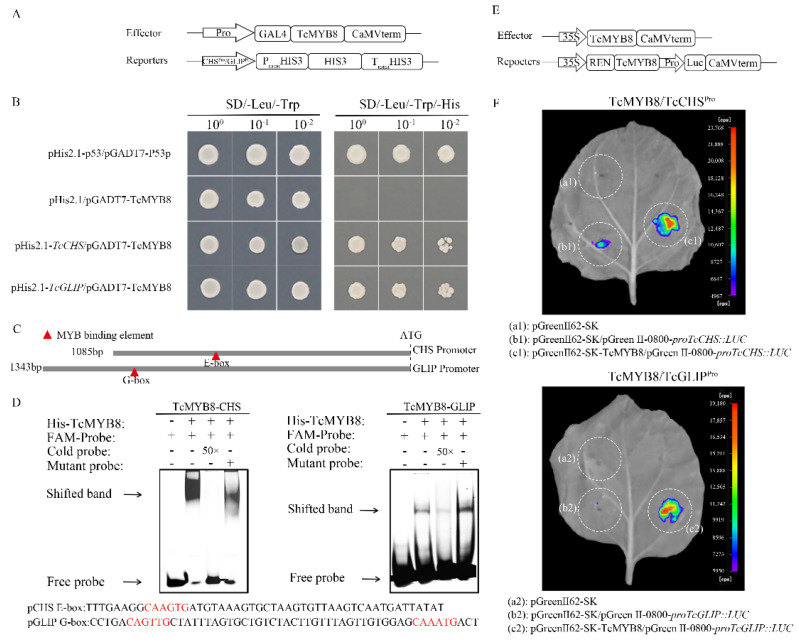
TcMYB8 directly binds to and activates the promoters of *TcCHS* and *TcGLIP*. (**A**) Schematic representation of the vectors used to perform Y1H assays. The proTcCHS/proTcGLIP was constructed into the pHis2.1 vector, while the *TcMYB8* was constructed into the pGADT7 vector. (**B**) Y1H assay showing that TcMYB8 binds to *proTcCHS/proTcGLIP*. Ten-fold serial dilutions of yeast cells carrying the constructs were plated on SD/-Leu/-Trp and SD/-Leu/-Trp/-His media. The pHIS2.1-p53 and pGADT7-p53 plasmids were used as positive controls. (**C**) Schematic diagram of E-box and G-box elements in the *proTcCHS/proTcGLIP*. (**D**) Electrophoretic mobility shift assay (EMSA) showing the direct binding of TcMYB8 to *proTcCHS/proTcGLIP*. The labeled probe (10 µM) was competed with a 50-fold excess of unlabeled wild-type cold probes and unlabeled mutant probes. The black arrows indicate shifted bands and free bands, respectively. (**E**) Schematic representation of the constructs used for transient expression assays. The *TcMYB8* gene was cloned downstream of the 35S promoter in the pGreen-62-SK vector to generate the effector construct, and the *proTcCHS/proTcGLIP* were separately cloned upstream of the *LUC* gene in the pGreen II-0800-*LUC* vector to generate the reporter constructs. (**F**) Transient expression assays showing that TcMYB8 binds to proTcCHS/proTcGLIP. (a1): pGreen-62-SK empty vector as the negative control, (b1): pGreen-62-SK/pGreen II-0800-*proTcCHS::LUC*, (c1): pGreen-62-SK-TcMYB8/pGreen II-0800-*proTcCHS::LUC*, (a2): pGreen-62-SK empty vector as the negative control, (b2): pGreen-62-SK/pGreen II-0800-*proTcGLIP::LUC*, (c2): pGreen-62-SK-TcMYB8/pGreen II-0800-*proTcGLIP::LUC*.

The *TcMYB8*, *TcCHS*, and *TcGLIP* genes showed similar expression profiles, suggesting that these genes potentially interact with each other. To test this possibility, we carried out yeast one-hybrid (Y1H) assays by constructing the required vectors (Figure 4A). The results showed that TcMYB8 binds to the promoters of *TcCHS* and *TcGLIP* genes (Figure 4B). Sequence analysis on the PlantMAN website revealed that the promoter sequences of *TcCHS* and *TcGLIP* contain a large number of MYB-binding sites (MBSs), E-box elements, G-box elements, and MYB core elements. The E-box elements in the pro*TcCHS* and the G-box elements and MBSs in the pro*TcGLIP* were labeled with the FAM fluorophore (Figure 4C). The purified TcMYB8 protein formed a shifted band with the labeled probe, and strong affinity was observed between the TcMYB8 protein and E-box probe. TcMYB8, as a 1R-MYB transcriptional activator, bound to the E-box (CAAGTG) and G-box (CAGTTG/CAAATG) motifs in the pro*TcCHS*, and pro*TcGLIP* genes, respectively, and activated their transcription (Figure 4D). A dual-luciferase (LUC) reporter assay showed that TcMYB8 effectively activated the expression of the *LUC* reporter gene when expressed under the control of pro*TcCHS* and pro*TcGLIP*, and played the role of a transcriptional activator by directly binding to the pro*TcCHS* and pro*TcGLIP* (Figure 4E,F). These results suggest that TcMYB8 activates the pro*TcCHS* and pro*TcGLIP*, and acts as a positive regulator of pyrethrin biosynthesis.

## 3. Discussion

### 3.1. TcMYB8, a 1R-MYB TF, Is a Novel MYB TF Found in T. cinerariifolium

MYB proteins are grouped into four classes (1R-, 2R-, 3R-, and 4R-MYB) based on the number of adjacent repeats (R) [32,33]. In the present study, we identified a novel 1R-MYB TF in *T. cinerariifolium*, TcMYB8, with a typical R3-type domain (primary structure: -F-X18-W-X18-W-) at the N terminus (Figure 1A). The 1R-type MYB TF genes, such as (*Arabidopsis*)*TRIPTYCHON* (*TRY*) and (*Actinidia eriantha*) *AceMYBS1*, are likely to have evolved from *R2R3-MYB* genes and are involved in epidermal cell patterning [34] and secondary metabolism control [35,36].

Among the flower and leaf expression patterns, the expression trends of *TcMYB8* were especially consistent with that of *TcCHS* and *TcGLIP* (Figure S2), suggesting that TcMYB8 may be a positive regulator of pyrethrin synthesis. In addition, pyrethrin content is affected by environmental factors, mechanical damage, and plant hormones [18,37,38,39], and, interestingly, we found elements responsive to these signals on the *proTcMYB8*. The expression of TcMYB8 was upregulated about 24-fold at 4 h after MeJA treatment compared with control tissues (Figure 2D). A recent article indicated that a single spray application of MeJA to *T. cinerariifolium* leaves rapidly upregulated the expression of most known pyrethrin biosynthesis genes and subsequently increased the total pyrethrin content in the leaf [40]. In addition, we found that JAZ4 (tify_33876) (Genbank accession no. ON961786), a JA-responsive protein, potentially interacts with TcMYB8 (Figure S3). These results imply that the regulation of pyrethrin synthesis by TcMYB8 in response to MeJA is a complex mechanism, and this process may have crosstalk with the jasmonate signaling pathway. Thus, our study provides a new insight into the pyrethrin regulatory network.

### 3.2. TcMYB8 Activates the Transcription of TcCHS and TcGLIP to Modulate Pyrethrin Content in T. cinerariifolium

Previous studies confirmed TcCHS and TcGLIP as key enzymes in the pyrethrin biosynthesis pathway (Figure S4) [18,19]. Three independent approaches (Y1H assay, dual-LUC assay, and EMSA) verified that TcMYB8 directly binds to the promoters of *TcCHS* and *TcGLIP* genes to upregulate their expression (Figure 4). Similar results have also been reported in *Chrysanthemum morifolium*, and CmMYB9a activates the biosynthesis of anthocyanins by positively regulating the expressions of *CmCHS*, *CmDFR*, and CmFNS [23]. Plant MYB TFs activate the transcription of downstream genes by binding to cis-elements, such as the G-box/E-box motifs and the MBSs in their promoter regions. *Tamarix hispida* ThMYB8 was found to bind to the MBSI motif to regulate the expressions of *ThCYP450-2*, *Thltk*, and *ThTIP* [41]. In citrus, (*Citrus clementina*) CiMYB42 was found to bind to the type II MYB core sequence (TTGTTG) in the *CiOSC* promoter to activate gene transcription [24]. In *Artemisia annua*, AaMYB16 and AaMYB5 exhibited significantly higher affinity for the L1-box element than that for the MBS [28]. In the current study, we verified that TcMYB8 could bind to the E-box and G-box cis-elements (Figure 4C,D).

In addition, we also transiently overexpressed or interfered with *TcMYB* gene expression in pyrethrum leaves to evaluate its impact on pyrethrins synthesis. It has been reported that in addition to the mainstream view of the synthetic pathway in glandular trichome (GT) cells, non-glandular trichome (non-GT) cells in *A. annua* plants could express the artemisinin biosynthetic pathway [42]. Interestingly, our previous research also found that the promoter of *TcCHS* can be expressed in mesophyll cells, and overexpression of *TcCHS* in mesophyll cells can increase pyrethrin content [19]. Instantaneous overexpression of TcMYB in mesophyll cells may directly activate the expression of *TcCHS* in mesophyll cells, thereby improving the ability of pyrethrin synthesis and significantly increasing the pyrethrins content. However, the effect of pTVR2-TcMYB8 treatment is not as significant as that of overexpression of *TcMYB* (Figure 3A,D) because it involves virus infection and plant defense response [43], and pyrethrin synthesis is a metabolic process involving a variety of tissues and organelles [6]. The influence of multiple factors may lead to the poor effect of *MYB*’*s* intervention. Therefore, a more accurate understanding of the effect of *TcMYB* in pyrethrum requires more appropriate experimental methods, such as CRISPR. However, the genetic transformation and gene editing of pyrethrum are still not mature enough.

### 3.3. How TcMYB8 Regulates Pyrethrin Biosynthesis

**Figure 5:**
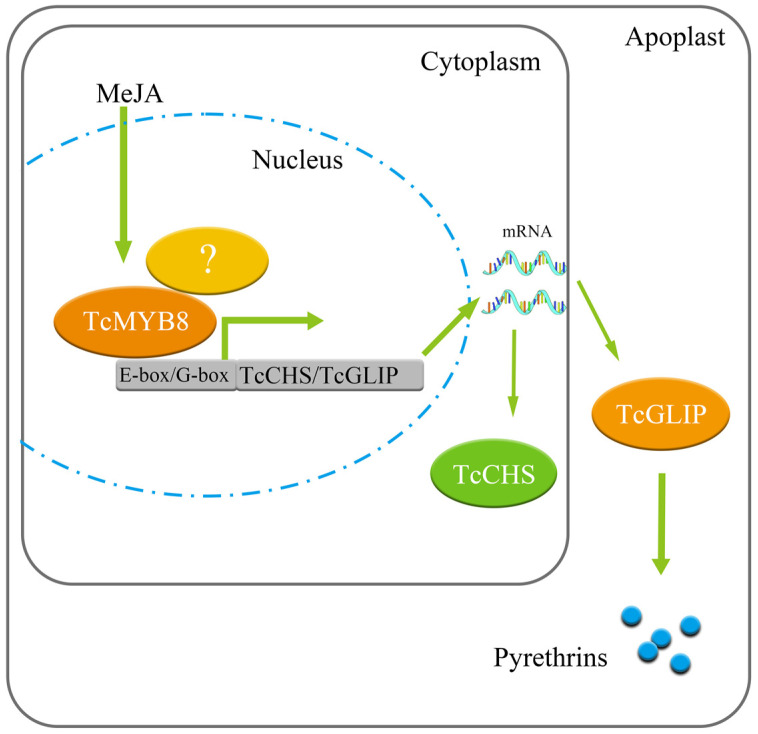
Working model for regulation of pyrethrin biosynthesis by TcMYB8. The white question mark in the figure indicates the tify33872 protein.

TcMYB8 is involved in the regulation of pyrethrin synthesis, and a complex regulatory network is formed among transcription factors, which makes the gene expression in pyrethrin biosynthesis more coordinated. The parallel regulatory relationships involved in the regulation of secondary metabolism are common, such as in *A. annua*, where WRKY, bHLH, and MYB TFs promote or inhibit the synthesis of artemisinin [44,45]. Jasmonate-ZIM-domain proteins interact with WD-Repeat/bHLH/MYB complexes and could regulate jasmonate-mediated anthocyanin accumulation and trichome initiation in *Arabidopsis thaliana* [46]. A recent report shows that *TcMYC2* (bHLH TFs) positively regulates pyrethrin biosynthesis [40]. Therefore, this study provides a new idea for WD-Repeat/bHLH/MYB complex and JA to participate in pyrethrin regulation (Figure 5).

## 4. Materials and Methods

### 4.1. Plant Materials, Growth Environment, and Phytohormone Treatments

Pyrethrum clone ‘W99′ obtained from the Key Laboratory for Biology of Horticultural Plants, Ministry of Education, Huazhong Agricultural University, Wuhan, China, was used in this study as it exhibits easy rooting, rapid growth, and high pyrethrin content. Using the clonal tissue culture seedlings of the same genotype for testing at a later stage can avoid the difference of pyrethrin content caused by different genotypes of *T. cinerariifolium*. Seedlings were grown in pots under greenhouse conditions (20 ± 5 °C and 12-h light/12-h dark photoperiod).

Different floral tissues were collected from *T. cinerariifolium* flowers at the S2 stage when half of the peripheral ray florets were open. Flowers at six different stages (S1–S6) and leaves in width at three different stages (L1–L3) were collected from field-grown plants, frozen in liquid nitrogen, and stored at −80 °C. One-month-old tissue culture seedlings of *T. cinerariifolium* were sprayed with 2 mM MeJA, 1 mM SA, or 1 mM ABA (prepared in 5 mL of 80% ethanol), and samples were collected at 0, 2, 4, and 8 h after hormone treatments.

### 4.2. RNA Extraction and Gene Expression Analysis

Total RNA was extracted from *T. cinerariifolium* using the TRIzol Reagent (Invitrogen, CA). Then, to synthesize first-strand cDNA, 1 µg of total RNA was reverse transcribed using the TransScript^®^ One-Step gDNA Removal kit and cDNA Synthesis SuperMix kit (TransGen Biotech, Beijing, China). Three biological replicates were performed for each gene, with each replicate containing two technical repeats [5]. *GADPH* was used as an internal reference gene [17]. Primers used for gene expression analysis are listed in Table S2.

### 4.3. Gene Cloning and Sequence Analysis

TcMYB8_ORF_F and TcMYB8_ORF_R primers were designed to amplify the ORF of TcMYB8. The relative molecular weight, isoelectric point, and stability of the TcMYB8 protein were predicted using ExPASy (http://web.expasy.org/protparam/ (accessed on 11 September 2020)). The amino acid sequences showing high homology to TcMYB8 were compared using DNAMAN software. A multiple sequence alignment was generated using ClustalW, and a phylogenetic tree was constructed using the MEGAX software with 1000 bootstrap replications [47]. The Genbank accession of the genes used in the assay were listed in Table S3. 

Promoter cloning was performed as described previously [48], and the reverse primers (R-SP1, R-SP2, and R-SP3) used to amplify the promoter of the *TcMYB8* gene are listed in Table S2. Cis-elements in the cloned promoter were predicted using the PlantCARE database (http://bioinformatics.psb.ugent.be/webtools/plantcare/html/ (accessed on 14 July 2022)).

### 4.4. Subcellular Localization Analysis

The *TcMYB8* CDS was amplified by PCR using sequence-specific primers containing *Swa*I and *Kpn*I restriction sites at the end. Using a ClonExpress^®^ II One Step Cloning Kit (Vazyme Biotech, Nanjing, China), the *TcMYB8* CDS (without the stop codon) was cloned into the pSuper1300-GFP vector (derived from pCambia130075) [49]. The obtained plasmid or empty vector (control) was introduced into *Agrobacterium tumefaciens* strain GV3101, which was then co-infiltrated into *N. benthamiana* leaves with the RFP-NLS plasmid (nuclear marker) [49]. After 72 h of shade adaptation, fluorescence signals were detected with a confocal laser scanning microscope (Leica TCS-SP8; Leica, Wetzlar, Germany).

### 4.5. Transient Overexpression of TcMYB8 in T. cinerariifolium Leaves

The transient gene expression assay was performed as previously described in chrysanthemum [49]. Briefly, *Agrobacterium* GV3101 (pSoup19) cells containing the pGreen-62-SK-TcMYB8 vector were inoculated into the YEB liquid medium containing 100 mg/L kanamycin, and cultured on a rotatory shaker at 28 °C for 12 h. The cells were harvested by centrifugation. The supernatant was removed, and the cells were resuspended in MES (containing 100 µM acetosyringone) to obtain a final optical density (OD_600_) of 0.6. The ground seedling leaves with unified genotype were taken, the bacterial solution was poured into a 500-mL beaker, and the vacuum was then applied at 0.23 atm for 5 min. The bacterial suspension was then discarded, and the leaves were transferred to a wet filter paper-lined Petri dish which was incubated in the dark for 4 days, as described previously [50]. After 4d of incubation, the agroinfiltrated leaf samples were used for qRT-PCR and HPLC analysis.

### 4.6. VIGS Assay

F-myb8-VIGS and R-myb8-VIGS primers (Figure S2) were designed to amplify the RNAi fragment of *TcMYB8*, which was then cloned into the *Bam*HI-digested pTRV2 vector by homologous recombination. The pTRV2 and pTRV1 plasmids were separately transformed into *Agrobacterium* GV3101 cells. *Agrobacterium* cultures containing the pTRV2 and pTRV1 plasmids were then co-infiltrated into *T. cinerariifolium* leaves.

### 4.7. Determination of Pyrethrin Content

The pyrethrins in the dried samples were extracted as described previously [9]. The sample was then filtered through a 0.22-µm membrane. Pyrethrin content was determined using the Waters HPLC system equipped with a photodiode array (PDA) detector using three biological replicates as described previously [51]. Pyrethrin standard (Sigma, Germany) was used for the identification of pyrethrin.

### 4.8. Y1H Assay

The *TcMYB8* ORF was cloned into the pGADT7 vector by homologous recombination, and the promoter sequences of *TcCHS* and *TcGLIP* were separately cloned into the pHis2.1 vector. The recombinant plasmid pGADT7:TcMYB8 was co-transformed with pHis2.1:*TcCHS* or pHis2.1:*TcGLIP* into the yeast strain Y187. The transformed yeast cells were selected on DDO (SD/-Leu/-Trp) medium and cultured on DDO (SD/-Leu/-Trp/-His) medium at 30 °C for 3 days to observe protein–DNA interaction (i.e., yeast growth).

### 4.9. Dual-LUC Reporter Assay

To construct the reporter plasmids, the promoter fragments of *TcCHS* and *TcGLIP* were separately cloned into the *Hin*dIII-linearized pGreenII-0800-LUC vector. To construct the effector plasmid, the CDS of *TcMYB8* was cloned downstream of the CaMV*35S* promoter in the *Hin*dIII-linearized pGreen-62SK vector. Introduction of the pGreen-62-SK empty vector and pGreen-62-SK/pGreenII-0800-LUC-proTcCHS and pGreenII-0800-LUC-proTcGLIP constructs together served as the negative controls. The resultant pGreen-62-SK-TcMYB8 vectors were transiently co-expressed in *N. benthamiana* leaves along with pGreenII-0800-LUC-*proTcCHS* or pGreenII-0800-LUC-*proTcGLIP*. Luminescence was detected using the LB985 system (Berthold, Bad Wildbad, Germany).

### 4.10. EMSA

The CDS of *TcMYB8* (without the stop codon) was cloned into the pET6HN-C vector, and the resultant plasmid was transformed into the *Escherichia coli* Rosetta (DE3) strain (Weidi, Shanghai, China). The strain was cultured in LB liquid medium containing 100 mg/L ampicillin until the OD_600_ value of approximately 0.6 was obtained. Protein induction was performed with 0.5 mM IPTG, followed by shaking at 18 °C for 16 h. The Ni NTA Resin (TransGen, Beijing, China) and a 30-kDa purification column (7500 rpm, 20–40 min, 4 °C) were then used to purify the protein.

The cis-elements (E-box motif, G-box motif, and MYB core sequence) in *TcCHS* and *TcGLIP* promoters were labeled with 5′6-FAM fluorophore, and 50-fold excess of unlabeled wild-type and cis-element mutant DNA fragments were used as competitors [52].

### 4.11. Statistics

All experiments were performed using at least three biological replicates. One-way ANOVA and Student’s t-test were performed using SPSS v.20 software. Significant differences were detected by Student’s t-tests, *p*-values < 0.05 were considered statistically significant, and error bars represent SE. In the figures, the following notations are used: *, *p* < 0.05 and **, *p* < 0.01.

## 5. Conclusions

TcMYB8 is the first MYB TF identified in *T. cinerariifolium*. TcMYB8 promotes pyrethrin biosynthesis by activating the expression of *TcCHS* and *TcGLIP* genes. This study reveals *TcMYB8* as a candidate gene for the breeding of pyrethrin-rich lines and for the genetic improvement of the *T. cinerariifolium* germplasm. In addition, this study also provides a foundation for the further analysis of the role of JA in the regulation of pyrethrin biosynthesis, and is expected to uncover the mystery of the mechanism underlying the dynamic balance of pyrethrin biosynthesis.

## Data Availability

Not applicable.

## Supplementary Materials

The following supporting information can be downloaded at: https://www.mdpi.com/article/10.3390/ijms232012186/s1.
