# Supplementary material for: TcMYB8, a R3-MYB Transcription Factor, Positively Regulates Pyrethrin Biosynthesis in Tanacetum cinerariifolium
Source: Int J Mol Sci. 2022 Oct 12;23(20):12186. doi: 10.3390/ijms232012186 (PMC9602545; doi:10.3390/ijms232012186)
Supplement: Supplementary file 1 [file ijms-23-12186-s001.zip › ijms-1927278-supplementary.pdf]

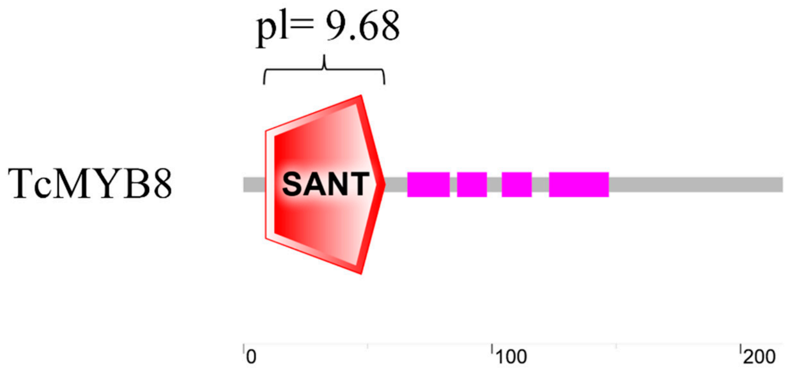

Figure S1. Conserved domain analysis of TcMYB8 protein. It contains one conserved MYB domain with a basic pI (9.68).

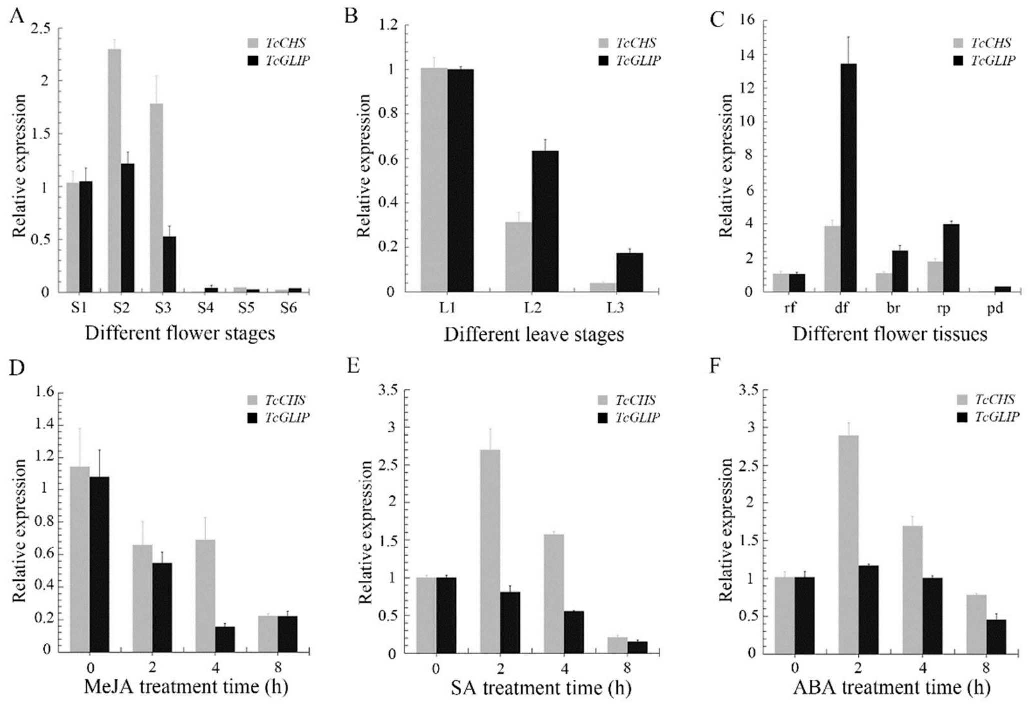

Figure S2. Expression patterns of *TcCHS* and *TcGLIP*. (A) qRT-PCR analysis of *TcCHS* and *TcGLIP* expression level in different stages of flowers (S1: bud, S2: half-open of peripheral ray flower, S3: 1st row of disk flower open, S4: half rows of disk flowers open, S5: all rows of disk flowers open, S6: past flowering); (B) qRT-PCR analysis of *TcCHS* and *TcGLIP* expression level in different stages of leaves, L1: small leaves (5-10mm in width), L2: medium leaves (15-20mm in width), L3: large leaves (>25mm in width; (C) qRT-PCR analysis of *TcCHS* and *TcGLIP* expression level in different flowers tissues (S2: ray flower, disk flower, bracts, receptacle and pedicel); (D) qRT PCR analysis of *TcCHS* and *TcGLIP* expression level within 8 hours under MeJA treatment of tissue culture seedlings; (E) qRT PCR analysis of *TcCHS* and *TcGLIP* expression level within 8 hours under SA treatment of tissue culture seedlings; (F) qRT PCR analysis of *TcCHS* and *TcGLIP* expression level within 8 hours under ABA treatment of

tissue culture seedlings.

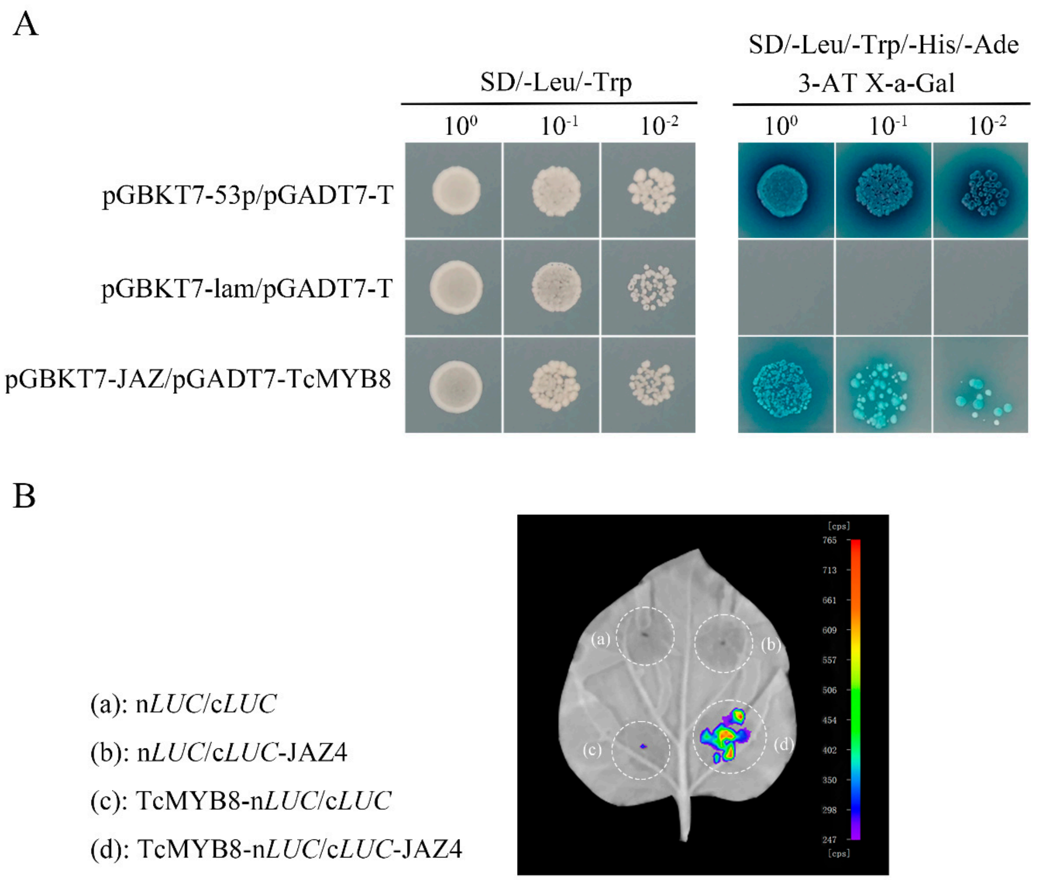

Figure S3. The interaction between TcMYB8 and tify_33876 (JAZ4) protein. (A) Y2H assay showing *TcMYB8* interacting with JAZ4; (B) Luciferase Report showing that TcMYB8 protein could interact with JAZ4. (a): nLUC/cLUC, (b): nLUC/cLUC-JAZs, (c): TcMYB8- nLUC/cLUC, (d): TcMYB8- nLUC/cLUC-JAZ4.

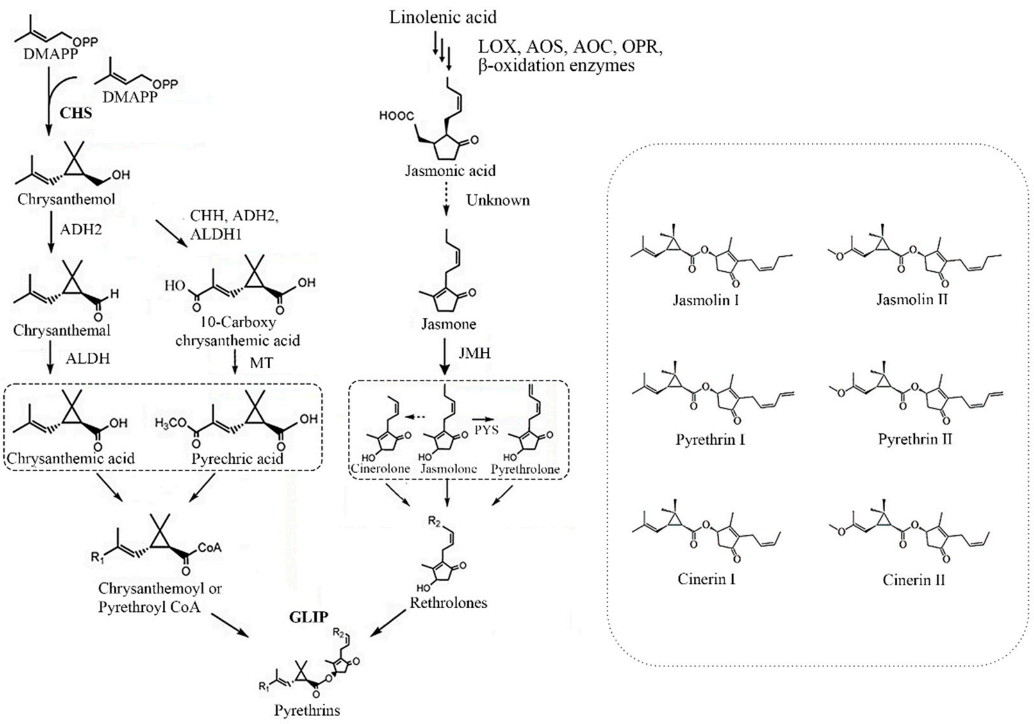

Figure S4. Pyrethrin metabolic pathway and pyrethrin components.

Table S1. Part of cis-acting regulatory elements in promoters of *TcMYB8*

Class	Name	Core Sequence	Num	Predictive Function
Transcriptional regulation	TATA-box	TATA	6	Core promoter element around -30 of transcription start
	CAAT-box	CAAT/CAAAT	15	Common cis-acting element in promoter and enhancer regions
Hormone response	GCCCORE	GCCGCC	1	Cis-acting regulatory element involved in the MeJA-responsiveness
	T/GBOXATPIN2	AACGTG	3	
	ABRE3a	TACGTG	1	Cis-acting element involved in the abscisic acid responsiveness
	ABRE4	CACGTA	1	
	ABRE	ACGTG	2	
	MYB2	TAACTG	1	
	MYCATRD22	CACATG	1	
	MYC	CATGTG	1	
	P-box	CCTTTTG	1	Cis-acting element involved in gibberellin-responsiveness
	TCA-element	CCATCTTTTT	1	Cis-acting element involved in salicylic acid responsiveness
	TCA-element	TCATCTTCAT	1	
	WBOXATNPR1	TTGAC	4	
	ELRE	TTGACC	1	Cis-acting element involved in ETH responsiveness
	TATCCAOSAMY	TATCCA	2	
Stress response	W-box	TTGACC	2	Trauma response
	E-box	CATGTG	1	Participating in phenylalanine metabolism elements
Light response	MRE	AACCTAA	1	MYB binding site involved in light responsiveness
	GATA-motif	GATAGGA	1	Part of a light responsive element
	TCT-motif	TCTTAC	2	
	GT1-motif	GGTTAA	1	Light responsive element
	G-Box	CACGTT	1	Cis-acting regulatory element involved in light responsiveness
	G-box	TACGTG	1	

Table S2. Primers used in experiments.

ID	Primer Name	Primer Sequences（5' to 3'）
Cloning	TcMYB8_ORF_F	ATGAACTATCTTCGGCCCGG
Cloning	TcMYB8_ORF_R	TCATACATTTTGATCTTCGCCG
Realtime PCR	TcCHS_RT_F	ACGTGCATCTTCTGGACCTCTTC
Realtime PCR	TcCHS_RT_R	TGAACAATCCGACGGTTAAGAGTC
Realtime PCR	TcGLIP_RT_F	GCCGGGAATGCGAGCAAAACAAC
Realtime PCR	TcGLIP_RT_R	CGCTCTCGCCTTCCTTAAAACCATA
Realtime PCR	TcGAPDH_RT_F	AAGGAGGAATCTGAAGGAAAGCTG
Realtime PCR	TcGAPDH_RT_R	GTTGTTGTTCAAAGCGATTCCAGC
Realtime PCR	GAPDH_RT_F	AAGGAGGAATCTGAAGGAAAGCTG
Realtime PCR	GAPDH_RT_R	GTTGTTGTTCAAAGCGATTCCAGC
Realtime PCR	TcMYB8_RT_F	TGCCAAGTCAACTAAAGTCAAACC
Realtime PCR	TcMYB8_RT_R	CGATTGCGATGAAGACGATGAAG
linking to pHis2.1 vector	CHS_Pro_pHis_F	gactcactatagggcgaattcGCTATTATAAAATCCCGTGTCTATGC
linking to pHis2.1 vector	CHS_Pro_pHis_R	attactagtggatccacgcgtCATTTACAACAGAATCTTAATGTGAGTGT
linking to pHis2.1 vector	GLIP_Pro_pHis_F	gactcactatagggcgaattcAAACTAGAAGCAAAGATCATCGTACTTC
linking to pHis2.1 vector	GLIP_Pro_pHis_R	attactagtggatccacgcgtAGCTTATATGTGCTCAGACAAGAGGT
linking to pGADT7 vector	MYB8_pGADT7_F	gtaccagattacgctcatatgATGAACTATCTTCGGCCCGG
linking to pGADT7 vector	MYB8_pGADT7_R	acgattcatctgcagctcgagTCATACATTTTGATCTTCGCCG
linking to pSuper1300GFP vector	MYB8_s1300g_F	gggcccggggtcgacatttaaatATGAACTATCTTCGGCCCGG
linking to pSuper1300GFP vector	MYB8_s1300g_R	gcccttgctcaccatggtaccTACATTTTGATCTTCGCCGTCTT
linking to pGreenIISK62 vector	MYB8_SK62_F	caggaattcgatatcaagcttATGAACTATCTTCGGCCCGG
linking to pGreenIISK62 vector	MYB8_SK62_R	gtcgacggtatcgataagcttTCATACATTTTGATCTTCGCCG
linking to pGreenII0800 LUC vector	CHS_LUC_F	gtcgacggtatcgataagcttGCTATTATAAAATCCCGTGTCTATGC
linking to pGreenII0800 LUC vector	CHS_LUC_R	caggaattcgatatcaagcttTTACAACAGAATCTTAATGTGAGTGTATGT
linking to pGreenII0800 LUC vector	GLIP_LUC_F	gtcgacggtatcgataagcttAAACTAGAAGCAAAGATCATCGTACTTC
linking to pGreenII0800 LUC vector	GLIP_LUC_R	caggaattcgatatcaagcttAGCTTATATGTGCTCAGACAAGAGGT

linking to pET6HN-C vector	MYB8_pET6N_F	gatctctaagcttgcgaattcATGAACTATCTTCGGCCCGG
linking to pET6HN-C vector	MYB8_pET6N_R	accaggcggccgccagaattcGCTACATTTTGATCTTCGCCG
Probes used in EMSA	Probe_proCHS _F	TTTGAAGGCAAGTGATGTAAAGTGCTAAGTGTTAAGTCAATGATTATAT
Probes used in EMSA	Probe_proCHS _R	ATATAATCATTGACTTAACACTTAGCACTTTACATCACTTGCCTTCAAA
Probes used in EMSA	Probe_proGLIP_F	CCTGACAGTTGCTATTTAGTGCTGTCTACTTGTTTAGTTGTGGAGCAAATGACT
Probes used in EMSA	Probe_proGLIP_R	AGTCATTTGCTCCACAACTAAACAAGTAGACAGCACTAAATAGCAACTGTCAGG
Probes used in EMSA	mProbe_proCHS _F	TTAAAAAGCAAAAAAATGTAAAGTGCTAAGTGTTAAAAAAAAAAATTATAT
Probes used in EMSA	mProbe_proCHS _R	ATATAATTTTTTTTTTAACACTTAGCACTTTACATTTTTTGCTTTTTAA
Probes used in EMSA	mProbe_proGLIP_F	CCTAAAAAAAACTATTTAGTGCTGTCTAAAAATTTAGTTGTGGAGAAAAAAACT
Probes used in EMSA	mProbe_proGLIP_R	AGTTTTTTTCTCCACAACTAAATTTTTAGACAGCACTAAATAGTTTTTTTTAGG
ptrv1_check_F	Ptrv1_f3807	GGCCTTGCGCCGTTCCAGAT
ptrv1_check_R	ptrv1_r4667	CCCAAAGGAAGGCCGCCCAC
ptrv2_check_F	ptrv2_f1604	TTATTACGGACGAGTGGACTTAG
ptrv2_check_R	ptrv2_r1761	AACTTCAGACACGGATCTACTT
linking to pTRV2 vector	MYB8_VIGS_F	agaaggcctccatggggatccCGATAGTTTCGATAGCGTAATGATG
linking to pTRV2 vector	MYB8_VIGS_R	cgtgagctcggtaccggatccTTGATCTTCGCCGTCTTCGA
chromosome walkin cloning	R-SP1	TAGTTTCGGACTCATCGAGCGGGT
chromosome walkin cloning	R-SP2	GGCCTTTGAGTTCCGTGGCTAT
chromosome walkin cloning	R-SP3	GGTGAAGCTTCCTCTCTTGATCCC

Table S3. Genbank accession of the genes used in the assay

Name	GenBank Accession	Species
HaMYB8	XP_022022105.1	*Helianthus annuus*
LsMYB8	XP_023745613.1	*Lactuca sativa*
CcMYB8-like	XP_024994176.1	*Cynara cardunculus* var. scolymus
EcMYB8-like	XP_043638944.1	*Erigeron canadensis*
HaMYB111	XP_021969240.1	*Helianthus annuus*
GhMYB9A	CAD87009.1	*Gerbera hybrid cultivar*
EcMYB6-like	XP_043638942.1	*Erigeron canadensis*
DzMYB308-like	XP_022757570.1	*Durio zibethinus*
HuMYB308-like	XP_021280377.1	*Herrania umbratica*
PaMYB8-like	XP_034933413.1	*Populus alba*
TcMYB308	XP_007051504.1	*Theobroma cacao*
PeMYB308-like	XP_011023154.1	*Populus euphratica*
PuMYB3	QLB38141.1	*Populus ussuriensis*
PtMYB308	XP_002320876.1	*Populus trichocarpa*
BpMYB7	QEE59996.1	*Betula platyphylla*
TcMYB8	OP087309	*Tanacetum cinerariifolium*
tify_33876	ON961786	*Tanacetum cinerariifolium*
